# Analysis of anti-osteoporosis function of chlorogenic acid by gene microarray profiling in ovariectomy rat model

**DOI:** 10.1042/BSR20180775

**Published:** 2018-08-31

**Authors:** Jun Min, Zhen Yuan, Qiao Zhang, Sijian Lin, Kai Wang, Jun Luo

**Affiliations:** 1 Department of Rehabilitation, The Third Affiliated Hospital of Nanchang University, Nanchang, Jiangxi 330000, China; 2 Department of Rehabilitation, The Second Affiliated Hospital of Nanchang University, Nanchang, Jiangxi 330000, China; 3 Department of Orthopaedic, The Second Affiliated Hospital of Nanchang University, Nanchang, Jiangxi 330000, China

**Keywords:** chlorogenic acid, gene array profile, MAPK pathway, osteoporosis

## Abstract

The aim of the present study was to clarify the effect of chlorogenic acid (CGA) on estrogen deficiency-induced osteoporosis based on micro-computed tomography (micro-CT) and potential mechanism of gene regulation via microarray profiling. Eighteen female Sprague–Dawley rats were divided randomly into sham-operated group, ovariectomy (OVX) plus saline vehicle group, and OVX plus CGA treatment group (CGA at 45 mg/kg/day). The loss of bone mass of the femoral metaphysis was evaluated by micro-CT to represent. Gene expression profiling was analyzed for bone marrow mesenchymal stem cells (BMSCs) of OVX and OVXT groups. Bioinformatics analysis was used to find the potential pathways regulated by CGA. OVX-induced osteoporosis could decrease femur bone mineral density (BMD), bone volume/tissue volume (BV/TV), trabecula number (Tb.N), and trabecular thickness (Tb.Th) and increased the trabecular separation (Tb.Sp) and structure model index (SMI) in the rats. Gene microarray profiling showed 121 differentially expressed genes in collected BMSCs between OVX and OVXT groups were identified with a threshold of a two-fold change and *P*<0.05. Kyoto Encyclopedia of Genes and Genomes (KEGG) was used to analyze the potential mechanism of CGA and we observed that many mitogen-activated protein kinase (MAPK) pathway associtated genes were altered, suggesting this pathway may play an important role. CGA improved bone quality by modifying the BMD and trabecular microarchitecture. Differential expression genes were screened by gene microarray profile and the results suggested MAPK pathway might participate in the process of OVX-induced bone remodeling.

## Introduction

Postmenopausal osteoporosis is a worldwide problem that affects more than 200 million people [1]. The main cause of the postmenopausal osteoporosis is the change in hormonal levels. Low estrogen can reduce bone resorption by inhibiting osteoclast development as well as inducing osteoclast apoptosis [2,3]. In addition, estrogen deficiency is also involved in bone remodeling and then leads to bone mineral density (BMD) loss, a phenomenon which associates with higher risks of fractures and disability [4–6]. Hormone replacement therapy (HRT) as well as antiresorptive or anabolic drugs have been widely used in the prevention and treatment of the postmenopausal osteoporosis. However, long-term HRT might give rise to severe side effects, such as impairment of the normal bone metabolism and atypical fractures [7].

Therefore, more researchers focussed on exploiting natural compounds which had fewer side effects to treat the osteoporosis [8,9]. Previous studies reported that Soy foods could partially reverse ovariectomy (OVX)-induced osteopenia in both rats and women, which had a similar effect to the traditional drug raloxifene [10,11]. The plant-derived compounds with antioxidant activities could prevent bone loss by promoting osteoclastic differentiation [12,13].

Chlorogenic acid (CGA) was the most abundant polyphenol in Chinese herb Du zhong and these polyphenolic compounds had exhibited osteoprotective activity [14–16]. CGA could be widely found in many other plants and had numerous biological effects, such as antiapoptosis, antiobesity, and antitumor activities [17]. In our previous study, we found that CGA could effectively promote the BMD via the Shp2/PI3K/Akt pathway in OVX rat models [18]. However, the potential mechanism of the effective genes or functional pathways remained uncertain.

In the present study, we verified that CGA could prevent OVX-induced osteoporosis in rats’ models based on our previous study. Gene microarray profile was used for searching different gene expression and effective signal pathway from BMCs samples of CGA-treated OVX rats. The mitogen-activated protein kinase (MAPK) pathway was found by Kyoto Encyclopedia of Genes and Genomes (KEGG) analysis, which might be worthy of study in the future.

## Materials and methods

### Animals and treatments

The study protocol was approved by the Institutional Animal Care and Use Committee of Nanchang University. Eighteen six-week-old Sprague–Dawley, specific-pathogen free, female rats (body weight (BW): 243.66 ± 3.89 g) were housed at 23–25°C (air conditioned) under a 12-h light/dark cycle, housed with the multiple standard rodent cages (545 × 395 × 200 mm) in a cage. Ethical approval for the use of animals in the present study was granted by the Animal Research Ethics Committee of China. During the experimental period, all rats were allowed free access to distilled water and standard rat chow. The stable baseline and circadian variation in temperature and activity were monitored everyday. Acclimatized rats underwent either bilateral laparotomy (sham, *n*=6) or bilateral OVX (*n*=12) after being anesthetized with an intraperitoneal injection with 3% sodium pentobarbital (5 mg/kg/day). After the surgical procedures, sham rats were fed similar to the OVX rats to minimize differences in BW between the two surgical groups. Next, the OVX rats were divided randomly into two treatment subgroups (*n*=6 per group): OVX plus saline vehicle (CMC-Na) and OVX plus 45 mg/kg/day CGA (≥gical groups), Sigma–Aldrich, U.S.A.), respectively (OVXT). CGA was administered orally through a commerce-made gastric tube and treatments started on day 2 post-OVX and continued for 12 weeks. The BWs of animals were recorded weekly during the experimental period.

### Assessment of BMD and bone micro-architecture

According to the previous method, BMD was calculated using the 2D total bone mineral content (t-BMC) of the measured area and reported as g/cm^3^ [19]. Each rat’s right femur was measured by dual-energy X-ray absorptiometry (DXA) with the Lunar Prodigy Advance system (GE Healthcare, U.S.A.) equipped with appropriate software for bone density assessment in small animal’s laboratory. After that, we selected three representative right femurs from each group to evaluate the trabecular micro-architecture of the femoral metaphysis by micro-computed tomography (micro-CT) (Scanco Medical, Zu¨rich, Switzerland). Selection of the representative samples was based on the median value of t-BMD of the respective group [20]. Because the trabecular bone is rich in the distal femur compared with the proximal and middle regions, scan was performed from the proximal growth plate in the distal direction (18 μm/slice) for every selected femur sample. The volume of interest (VOI) was selected as a cross-sectional area spanning 100 slices from the proximal growth plate. This scanning generated 3D images of the micro-architecture that were examined and displayed. Bone morphometric parameters obtained from the CT, including the bone volume fraction (bone volume/tissue volume (BV/TV)), trabecular number (Tb.N), trabecular separation (Tb.Sp), trabecular thickness (Tb.Th), and structure model index (SMI), were analyzed for the VOI. The operator conducting CT analysis was blinded to the treatments associated with the specimens. All examinations were conducted according to the principles and procedures described in the most recent National Research Council publication of the Guide for the Care and Use of Laboratory Animals and referred to the ARRIVE guidelines [21].

### Isolation of rat bone marrow mesenchymal stem cells

All animal experiments complied with the animal protocols approved by the Institutional Review Board of the Second Affiliated Hospital of Nanchang University. Both femurs and tibias were dissected. Bone marrow cells were flushed out with PBS in a 5-ml syringe fitted with a gauge needle. Mononucleated cells were isolated by density gradient centrifugation in rat lymphocyte separation medium (Solarbio, Beijing, China) at a concentration of 1.091 g/ml. Isolated cells were seeded in standard low-glucose Dulbecco’s modified Eagle’s medium (DMEM, HyClone, Shanghai Bioleaf Biotech Co., Ltd., China), supplemented with 15% FBS (HyClone, Shanghai, China), and then cultured in a humidified incubator with 5% CO_2_ at 37°C. Then CD33 and CD44 marker was used to identify the bone marrow mesenchymal stem cells (BMSCs) and the specimens were sent to gene assay profile to find the differentially expressed genes.

### Gene microarray processing and quantitative RT-PCR validation

Microarray hybridization, scanning, and analysis were performed by Shanghai Biotechnology Corporation (Shanghai, People’s Republic of China). Briefly, each slide was hybridized with 1.65 μg Cy3-labeled cRNA using Gene Expression Hybridization Kit (Catalog# 5188-5242, Agilent Technologies, Santa Clara, CA, U.S.A.) in Hybridization Oven (Catalog# G2545A, Agilent Technologies, Santa Clara, CA, U.S.A.), according to the manufacturer’s instructions. After 17 h of hybridization, slides were washed in staining dishes (Catalog# 121, Thermo Shandon, Waltham, MA, U.S.A.) with Gene Expression Wash Buffer Kit (Catalog# 5188-5327, Agilent Technologies, Santa Clara, CA, U.S.A.), following the manufacturer’s instructions. Slides were scanned by Agilent Microarray Scanner (Catalog# G2565CA, Agilent Technologies, Santa Clara, CA, U.S.A.) with default settings, dye channel: green, scan resolution = 3 μm, PMT 100%, 20 bit. Data were extracted with Feature Extraction software 10.7 (Agilent Technologies, Santa Clara, CA, U.S.A.). Raw data were normalized by Quantile algorithm, limma packages in R. For comparison analysis, a two-class unpaired method in the Significant Analysis of Microarray software was applied to identify significantly differentially expressed genes between the two selected groups.

#### Statistical analysis of real-time PCR data

Differentially expressed genes were identified by statistically significant differences (*P*<0.05) in the microarray probe sets between the groups using the Mann–Whitney nonparametric test. Hierarchical cluster analysis was performed on each comparison to assess correlations amongst samples for each identified gene set. Initially identified candidate genes from the microarray were further assayed by RT-PCR analysis. Briefly, total RNA was extracted using TRIzol reagent (Catalog# 15596-018, Life Technologies, Carlsbad, CA, U.S.A.) following the manufacturer’s instructions and checked for a RIN number to inspect RNA integrity by an Agilent Bioanalyzer 2100 (Agilent Technologies, Santa Clara, CA, U.S.A.). Qualified total RNA was further purified by RNeasy mini kit (Catalog# 74106, Qiagen, GmBH, Germany) and RNase-free DNase set (Catalog# 79254, Qiagen, GmBH, Germany). All PCR assays were repeated three times. For RT-PCR results, the relative amount of expressed mRNA was calculated by comparison with the expression of the housekeeping gene in the same sample. Using the 2^−ΔΔ*C*^_t_ method, we present the data as the fold change in gene expression normalized and relative to the expression in the control group.

### KEGG analysis

The access to KEGG is through the GenomeNet website at http://www.genome.ad.jp/kegg/ [22]. The SOAP server is used for computerized access to KEGG at http://www.genome.ad.jp/kegg/soap/.

### Statistical analysis

Data were analyzed using SPSS software from IBM (version 13.0, U.S.A.). Student’s *t*test and Fisher’s exact test were used, as appropriate. Results were expressed as mean ± S.D. and a *P*-value <0.05 was considered to indicate statistical significance.

## Results

### Treatment with CGA induces the bone remodeling via micro-CT analysis

**Figure 1:**
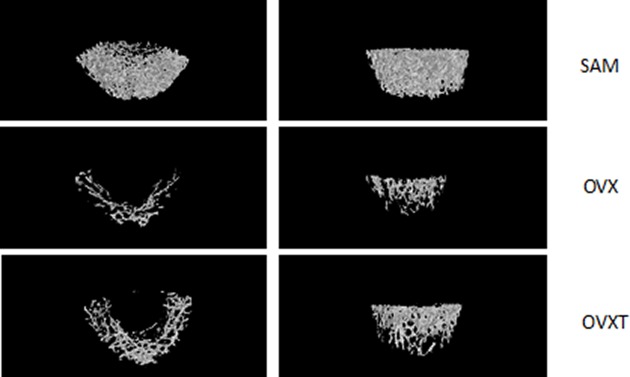
Effects of CGA on parameters of bone micro-architecture in rats Micro-CT images showing representative trabecular bone micro-architecture of the femoral metaphysis for each treatment group (first line: SAM group; second line: OVX group; third line: OVXT group).

Three-dimensional images of femoral metaphyses generated by micro-CT showed differences in trabecular micro-architecture amongst the various treatment groups (Figure 1). Analysis of data from the representative samples indicated that OVX decreased trabecular BV/TV (Sham: 19.97 ± 3.40%; OVX: 2.70 ± 0.33%), Tb.N (Sham: 2.32 ± 0.44 mm; OVX: 0.27 ± 0.01 mm), and Tb.Th (Sham: 0.08 ± 0.01 mm; OVX: 0.02 ± 0.01 mm) (all *P*<0.05), compared with the parameters obtained for the sham group. By contrast, SMI (Sham: 1.73 ± 0.22; OVX: 2.66 ± 0.08, *P*<0.05) in the proximal femur was increased in response to OVX. In addition, BMD was decreased (Sham: 0.28 ± 0.04 g/mm^3^; OVX: 0.07 ± 0.01 g/mm^3^; *P*<0.05). Then, all of these OVX effects were partially reversed in OVXT groups ((BV/TV (OVX: 2.70 ± 0.33%; OVXT: 7.20 ± 0.22%), Tb.N (OVX: 0.27 ± 0.01 mm; OVXT: 0.50 ± 0.09 mm), and Tb.Th (OVX: 0.02 ± 0.01 mm; OVXT: 0.04 ± 0.01 mm), SMI (OVX: 2.66 ± 0.08; OVXT: 2.19 ± 0.04), BMD (OVX: 0.07 ± 0.01 g/mm^3^; OVXT: 0.14 ± 0.02 g/mm^3^), all *P*<0.05 compared with OVX group) (Table 1).

**Table 1:** The parameters of rats’ femur micro-architecture and BMD between OVX and sham group

Variables	Groups		
	Sham	OVX	OVXT
Total VOI volume (TV, mm^3^)	13.08 ± 1.98	14.67 ± 1.12	16.36 ± 0.56
Object volume (BV, mm^3^)	2.31 ± 0.14	0.40 ± 0.08^1^	1.18 ± 0.07^2^
Percent object volume (BV/TV, %)	19.97 ± 3.40	2.70 ± 0.33^1^	7.20 ± 0.22^2^
SMI	1.73 ± 0.22	2.66 ± 0.08^1^	2.19 ± 0.04^2^
Tb.Th (mm)	0.08 ± 0.01	0.02 ±0.01^1^	0.04 ± 0.01^2^
Tb.N (1/mm)	2.32 ± 0.44	0.27 ± 0.10^1^	0.50 ± 0.09^2^
BMD (g/mm^3^)	0.28 ± 0.04	0.07 ± 0.01^1^	0.14 ± 0.02^2^

1 *P*<0.05 between OVX and sham groups.

2 *P*<0.05 between OVX and OVXT groups.

### Identification of genes expression in BMSCs through microarray profile and validated by real-time PCR

**Figure 2:**
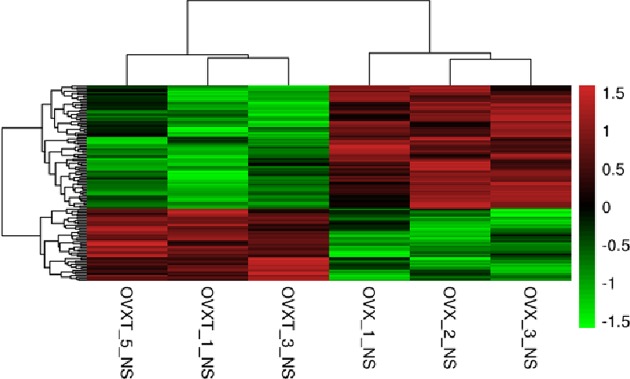
The heat map of differential expression of genes in BMSCs samples identified between OVX/OVXT group A total of 36 genes were found to be specifically expressed as the fold changes in the regulated genes were set as ≥2.0 or ≤0.5. Pseudocolors indicate differential expression (red, up-regulation; green, down-regulation; black, no expression change).

**Figure 3:**
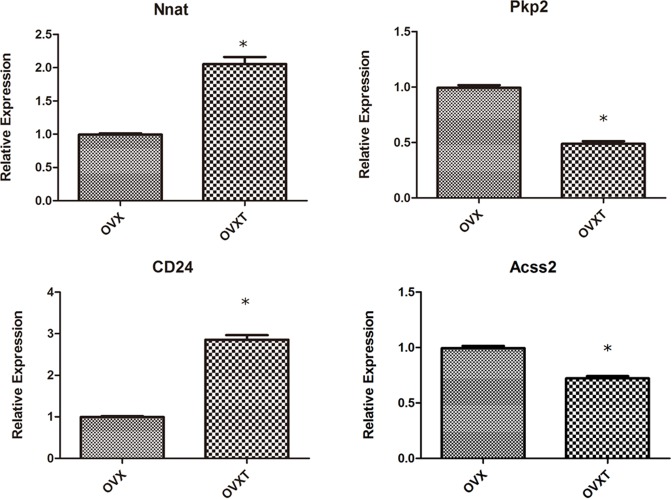
Qauntitative PCR analysis of four genes specifically expressed from BMSCs of OVX group compared with OVXT group (CGA treatment) Changes in expression observed by real-time quantitative-PCR agreed favorably with the microarray data.

Using Affymetrix GeneChip HG-U133 Plus 2.0, we analyzed global gene expression changes in BMSCs samples from six rats (three from OVX group and three from OVTX group). Genes were identified as being significantly regulated between groups when the unpaired *t*tests corrected for multiple comparisons had a false discovery rate ≤5%. A total of 121 genes were identified as differentially expressed between OVX and OVXT groups (fold change >2 and *P*<0.05) (Figure 2 and Supplementary Table S1). Amongst them, the top five most differential mRNAs were RGD1564664, Pdlim3, Myh11, Ear11, and Nnat Four genes (Acss2, Cd24, Nnat, and Pkp2) were further screened by fluorescence quantitative PCR to validate the microarray result. For all the four genes, changes in expression observed by RT-PCR agreed favorably with the microarray data (Figure 3).

**Figure 4:**
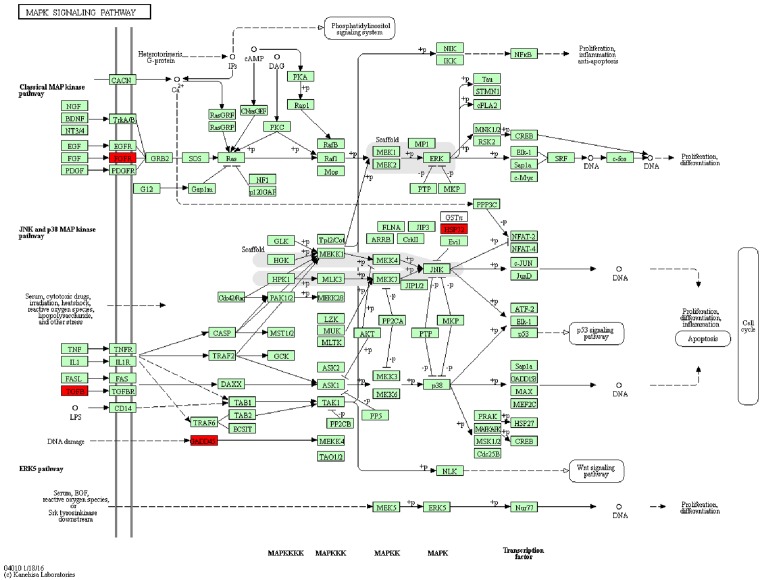
The MAPK signaling pathway of potential functional genes selected from BMSCs in OVX/OVXT group

### Analysis of KEGG and MAPK signal pathway

**Figure 5:**
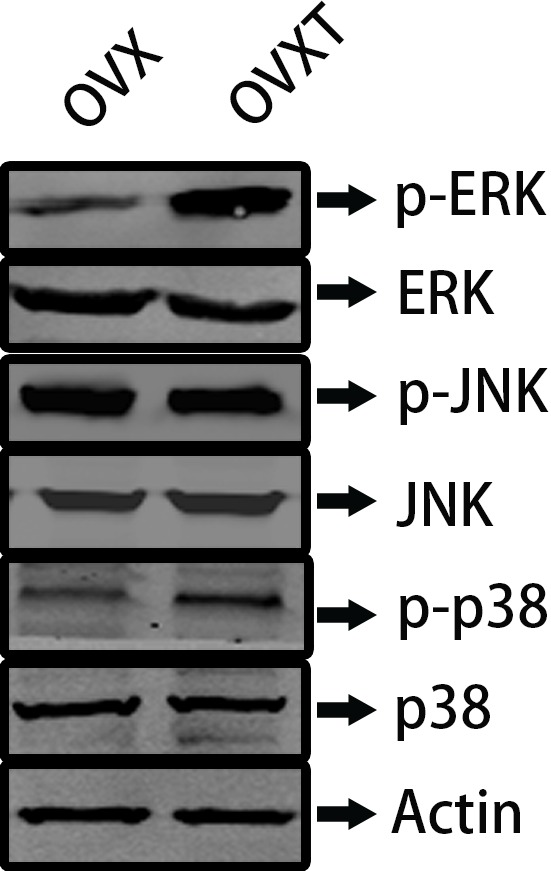
The comparison of phosphorylation levels of ERK, p38, and JNK between OVX and OVXT groups ERK, extracellular regulated protein kinases; JNK, c-Jun N-terminal kinase

KEGG is a bioinformatics resource for understanding higher order functional meanings and utilities of the cell or the organism from its genome information. According to general gene expression profiling in the present study and network searching in official KEGG websites, five genes including *Hspa1a, Fgfr2, Gadd45a, Tgfb3*, and *Hspa1b* were mapped on MAPK pathway and considered worth to further analyze potential functional effects on bone remodeling (Supplementary Figure S1 and Table S2). Next, we chose the classical proteins of MARK pathway to validate the bioinformatics results and we found that phosphorylations of ERK and p38 was increased in the OVXT group compared with the OVX group (Figure 5).

## Discussion

In the present study, we evaluated the specific parameters of femur micro-architecture to identify the CGA’s osteogenic effects in rats [21]. As expected, OVX resulted in a decreased femur BMD after 12 weeks and loss of bone mass was accompanied by a significant increase in bone remodeling. Although inhibition of bone remodeling would be considered beneficial, the bone function might be decreased if bone remodeling was inhibited for an excessive period of time. For instance, treatment with pamidronate (14 mg/kg/day for 25 days) could decrease intrinsic diaphyseal bone strength in rats [24]. Preservation of the trabecular micro-architecture contributed to bone strength and might reduce fracture risk [25–27]. The SMI was used to distinguish between rods and plates in trabecular bone. Analysis of our results indicated that there was a significant transition of trabecular structures from rods to a mixed plates-and-rods by OVX. However, CGA treatment could partially restore the trabecular bone [28].

Global gene expression profiling can be used to explore functional gene pathways. In the current study, we used global gene transcription analysis to identify gene changes in BMSCs derived from OVX and OVXT groups. A total of 36 genes with a threshold of a two-fold change and *P*<0.05 have been identified. We further validated four genes (Acss2, Cd24, Nnat, and Pkp2) by RT-qPCR and the similar trends suggested the results of global gene expression profiling were reliable.

Next, we found that *Hspa1a/Fgfr2/Gadd45a/Tgfb3/Hspa1b* genes were mapped on MAPK pathway by KEGG analysis. Previous study reported that CGA could affect the expression of apoptosis-related genes that were part of oxidative stress and p38 MAP-dependent pathways [29]. CGA could also counteract AP-induced liver injury by preventing apoptosis and oxidative stress damage, and more specifically, the MAPK signaling cascade appeared to be engaged in this protective mechanism [30]. Bone remodeling is also an important course during antiosteosis process which may be regulated by MAPK pathway. Strontium could promote osteogenic differentiation of MSCs through activating the Ras/MAPK signaling pathway and the downstream transcription factor Runx2 [31]. A similar study pointed out that sesamin had the ability to trigger osteoblast differentiation by activation of the p38 and ERK-MAPK signaling pathway and possibly indirectly regulated osteoclast development via the expression of OPG and RANKL in osteoblasts [32]. MAPK signaling pathway also might be involved during bone mechanism process. Researchers indicated strain-induced osteogenic differentiation of OVX BMSCs might be regulated by ERK1/2-MAPK signaling pathway [33]. In this study, we found that CGA administration in the OVX group could involve in the MARK signaling pathway by regulating the phosphorylation of p38 and ERK.

In our previous study, we induced the BMSCs to differentiate to the osteoblast and we found that CGA could prevent osteoporosis by Shp2/PI3K/Akt pathway [18]. However, we only used BMSCs as our models and our goal was to identify the gene changes in BMSCs. The limitation of our work was that we assumed that the changes of gene expression in BMSCs could directly regulate the anti-osteoporosis function, which needed to be validated by more experiments in the further investigations.

In summary, we found that CGA could effectively counteract osteoporosis in OVX rat models. Microarray results combined with bioinformatics analysis suggested that the MAPK pathway might be valuable for the mechanism research in future.

## Supporting information

**supplementary Figure:** 

**Supplemental Table:** 

**Supplemental Table:** 

## Abbreviations

Akt: protein kinase B
AP: acetaminophen
BMD: bone mineral density
BMSC: bone marrow mesenchymal stem cell
BV/TV: bone volume/tissue volume
BW: body weight
CGA: chlorogenic acid
CMC-Na: saline vehicle
HRT: hormone replacement therapy
KEGG: Kyoto Encyclopedia of Genes and Genomes
MARK: mitogen-activated protein kinase
micro-CT: micro-computed tomography
OPG: osteoclastogenesis inhibitory factor
OVX: ovariectomy
OVXT: ovariectomy plus CGA treatment
PI3K: phosphoinositide 3-kinase
PMT: a parameter in the microarray scanner
RANKL: receptor activator of nuclear factor kappa-B ligand
RIN: RNA integrity number
RT-PCR: reverse transcription-polymerase chain reaction
shp2: SH domain-containing protein tyrosine phosphatase 2
SMI: structure model index
Tb.N: trabecular number
Tb.Th: trabecular thickness
VOI: volume of interest
