# Supplementary figures and images for: Analysis of anti-osteoporosis function of chlorogenic acid by gene microarray profiling in ovariectomy rat model
Source: Biosci Rep. 2018 Aug 31;38(4):BSR20180775. doi: 10.1042/BSR20180775 (PMC6117622; doi:10.1042/BSR20180775)

# Top 30 of Pathway Enrichment

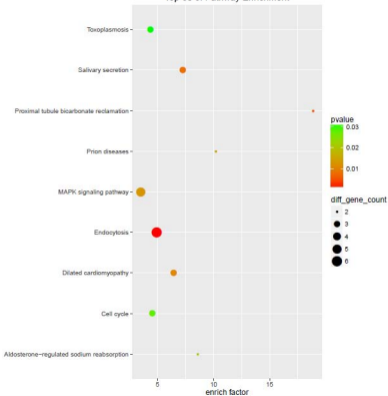

Supplement: Supplementary file 1 [file bsr20180775_Supp1.pdf]
